# Robot-assisted thoracoscopic surgery for large apical schwannoma in an obese patient

**DOI:** 10.1186/s44215-022-00007-0

**Published:** 2022-10-10

**Authors:** Shunta Ishihara, Masanori Shimomura, Hiroaki Tsunezuka, Satoshi Ikebe, Masayoshi Inoue

**Affiliations:** 1 Division of Thoracic Surgery, Department of Surgery, Graduate School of Medical Science, Kyoto Prefectural University of Medicine, 465 Kajii-cho, Kamigyo-ku, Kyoto, 602-8566 Japan; 2 Department of Thoracic Surgery, Otsu City Hospital, 2-9-9 Motomiya, Otsu, Shiga 520-0804 Japan

**Keywords:** RATS, Robotic surgery, Thoracic apex, Obese, Sschwannoma, Mediastinal tumor, Robotics, Surgery/incisions/exposure/techniques/ Thoracic outlet

## Abstract

**Background:**

Neural tumors at the thoracic apex require a careful surgical technique because of the presence of vascular and neural structures and minimally invasive surgery can be challenging for these tumors, especially large tumor in obese patients. We report a case of a large apical schwannoma that treated with RATS in an obese patient.

**Case presentation:**

An obese, diabetic, 36-year-old obese man with a body mass index of 34.7 presented with a 5.0-cm mediastinal mass in the left thoracic apex diagnosed via chest computed tomography. The magnetic resonance images suggested a schwannoma. Surgery was scheduled after glycemic control. Robot-assisted thoracoscopic surgery (RATS) was performed with in the right lateral position, and tumor was dissected from the surrounding tissue without severe nerve injury using bipolar forceps. The pathological diagnosis was benign schwannoma. The patient had an uneventful clinical course and was discharged on postoperative day 3.

**Conclusion:**

RATS may be a useful approach in the narrow space of the thoracic cavity in obese patients.

**Supplementary Information:**

The online version contains supplementary material available at 10.1186/s44215-022-00007-0.

## Background

The apex of the thoracic cavity contains the subclavian vessels, brachial plexus, and sympathetic nerve trunk and requires a careful surgical technique and the approach. Mediastinal tumors on the thoracic apex can be safely operated on via thoracotomy with an anterior or posterior approach, but minimally invasive approach should be considered in the case of benign schwannoma. Video-assisted thoracoscopic surgery (VATS) can safely remove apical neurinomas and preserve nerve function [1]. However, a large tumor in the thoracic apex would be difficult to dissect from adjacent structures using VATS due to the linear forceps. Robot-assisted thoracoscopic surgery (RATS) has been reported to be a good option for mediastinal tumors in narrow spaces [2]. We report a case of a large apical schwannoma that treated with RATS.

### Case presentation

**Fig. 1:**
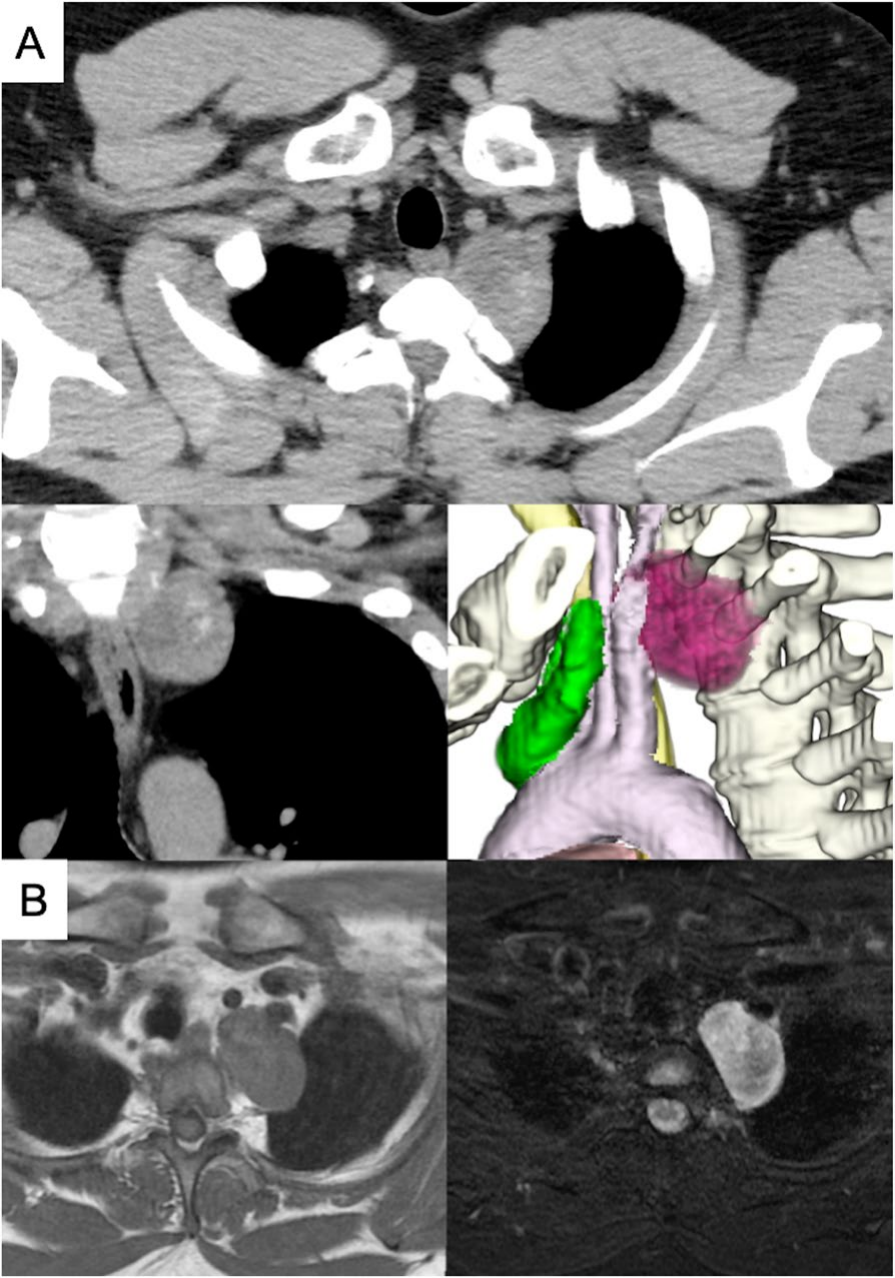
**A** Chest computed tomography (CT) shows a 5.0-cm mass in the thoracic apex. The tumor is adjacent to the left subclavian vessels, common carotid artery, and first vertebral body. **B** Magnetic resonance images show T1 iso intensity and T2 high intensity

**Fig. 2:**
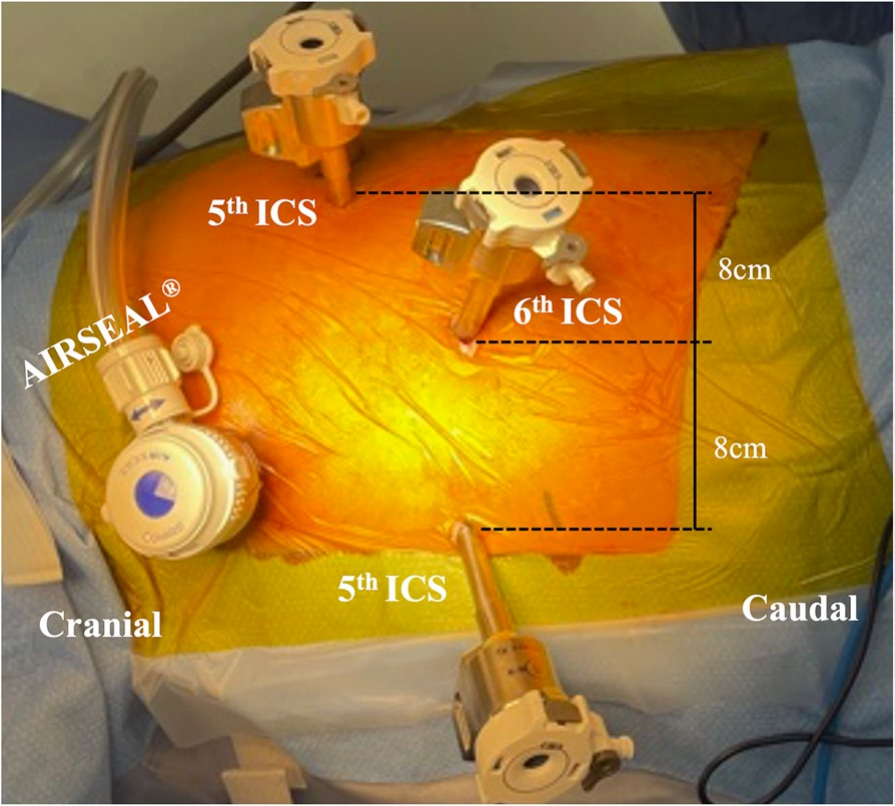
Port placement for robot-assisted tumor resection. The distance of between ports is 8.0 cm

**Fig. 3:**
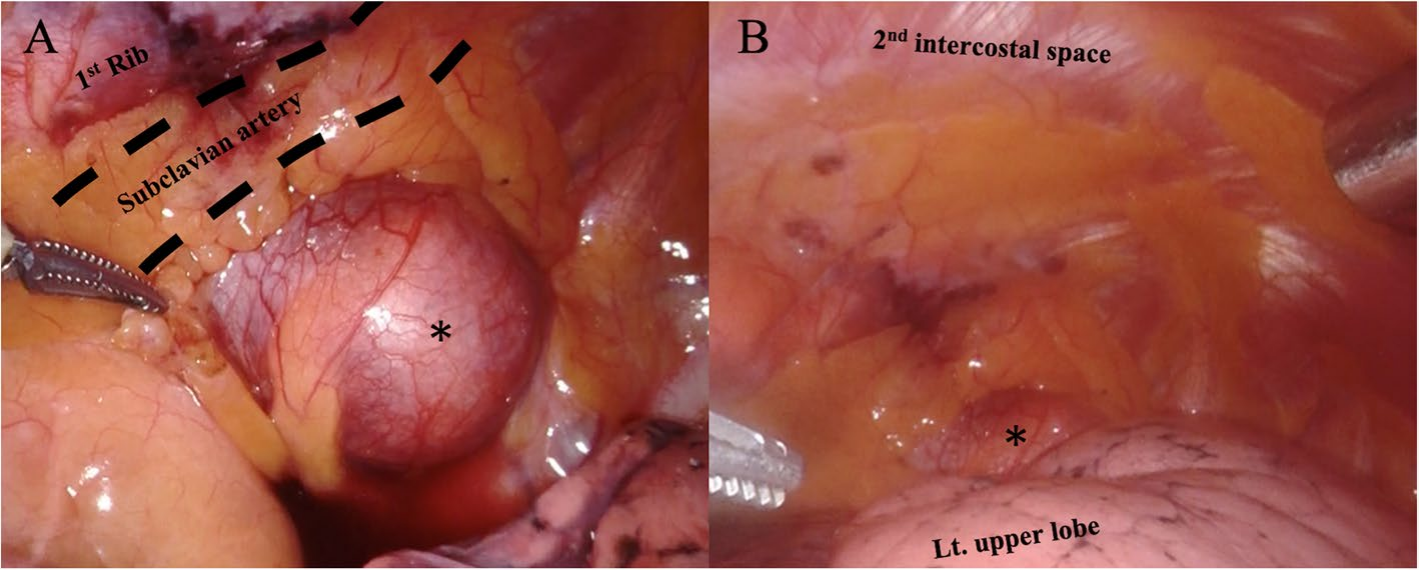
**A** Intraoperative photographs of the left superior sulcus tumor (star) adjacent to the left subclavian artery. **B** General view of the apex of the thoracic cavity

A 36-year-old man with a high body mass index of 34.7 (height = 173 cm and weight = 103.9 kg) was diagnosed with a mediastinal mass on chest roentgenogram. Chest computed tomography (CT) revealed a mass measuring 5.0 cm in the superior sulcus adjacent to the left subclavian vessels, common carotid artery, first rib, and brachial plexus (Fig. 1A). Magnetic resonance imaging showed T1 low intensity and T2 high intensity and was suggestive of schwannoma (Fig. 1B). The patient had a history of type 2 diabetes mellitus and a high HbA1c level (HbA1c = 9.1%). Surgery was scheduled under glycemic control for the diabetologist. RATS was performed in the right lateral position. An operating arm was placed at the mid-clavicular line of the 6th intercostal space (ICS) for the camera-port. The operative port was placed at the mid-clavicular line of the 5th ICS and at the anterior border of the scapula of the 5th ICS. An assistant port was placed at the anterior axillary line of the 3rd ICS (Fig. 2). Insufflation of CO_2_ was performed through the assistant port to a pressure of 8 mmHg. The tumor was located at the apex of the thoracic cavity, adjacent to the subclavian artery (Fig. 3). The left arm of the surgical system was equipped with long bipolar grasper (Endo Wrist; Intuitive Surgical), and the right arm initially had a permanent cautery spatula (Endo Wrist; Intuitive Surgical) used for dissection of the pleura covering the tumor, which was later replaced with a Maryland bipolar forceps (Endo Wrist; Intuitive Surgical) to dissect the surrounding tissue and vessels. The tumor extended along the sympathetic trunk. The console and the operation times were 84 min and 137 min, respectively. The estimated blood loss was 3 g. The postoperative course was uneventful, and a mild drooping left eyelid was noted on physical examination on postoperative day one. The chest tube was removed the day after surgery, and the patient was discharged on postoperative day 3. The final pathological diagnosis of the mediastinal tumor was a benign schwannoma.

### Discussion

Because of the presence of adjacent critical structures, safe operation for superior sulcus tumors requires open thoracotomy. However, for benign schwannomas, open thoracotomy leads to be highly invasive, and the approach to surgery for the schwannomas in the thoracic apex is controversial. Endo et al. reported that VATS approach was useful for the superior sulcus and neurinomas [1]; however, tumors larger than 3.5 cm were selected thoracotomy. On the apex of the thoracic cavity, the linearity of the forceps interferes with each other used in VATS, limiting manipulation. Especially in large apex tumors, the backside of the tumor with a blind spot makes dissection from critical structures difficult. RATS can perform precise manipulation using articulation [3], which allows the procedure to be performed in a circular fashion, making it safer to perform delicate manipulations. Inferior mediastinal tumors are similarly difficult to visualize, even with thoracoscopy, and Fukui et al. reported that robot-assisted surgery is useful for tumors in narrow spaces surrounding the vertebral body and the descending aorta by devising a port arrangement. [4] RATS may be useful for surgery in areas where surgical challenges are anticipated with the conventional thoracoscopic approach.

In the thoracic apical tumors, nerve damage is the most common postoperative complication. Because of the presence of the diaphragmatic nerve, vagus nerve, brachial plexus, and sympathetic nerve trunks in the apex of the thoracic cavity, it is recommended to avoid the dissection using monopolar electrocautery [5]. Monopolar electrocautery is more likely to cause nerve damage as the current flows to deeper tissues, while bipolar electrocautery minimizes nerve damage as the current only flows through the tissue between the two electrodes. As schwannomas are adjacent to nerves, electrocautery should be used carefully. In this case, perineural manipulation was performed using Maryland-bipolar forceps. In thoracic apex tumors, a bipolar system of forceps such as the one used in this case may be useful for preventing nerve damage.

In obese patients, the depth of the body cavity limits the use of instruments, leading to longer operative times and increased postoperative complications [6]; therefore, minimally invasive surgery should be selected when dealing with benign tumors in order to reduce surgical stress. RATS for obese patients with non-small cell lung cancer is reported to be feasible and safe, with significant advantages when compared to open surgery in terms of early postoperative outcomes [7]. In minimally invasive surgery, RATS shows little difference in surgical outcomes, including survival and complications compared to VATS [8]. In obese patients, RATS has been shown to have a lower rate of conversion to thoracotomy and fewer complications of respiratory failure than VATS, because RATS has advantage on the less thoracic space [9]. In the present case, it was possible to remove the chest drain the day after surgery, and the patient discharged early without complications.

Robotic surgery may be advantageous in obese patients with large benign apical tumors compared to the conventional VATS or open thoracotomy.

## Supplementary Information

**Additional file 1.** 

## Data Availability

Not applicable.

## Abbreviations

RATS: Robot-assisted thoracoscopic surgery
VATS: Video-assisted thoracoscopic surgery
CT: Computed tomography
ICS: Intercostal space
